# Therapeutic potential of TRIM family proteins in gastric cancer: from signaling pathway regulation to precision targeting strategies

**DOI:** 10.3389/fonc.2025.1628729

**Published:** 2025-08-27

**Authors:** Caiqing Zhao, Jialing Qi, Zhenze Zhang, Qi Feng, Ziling Fang, Nian Fang

**Affiliations:** ^1^ Department of Gastroenterology, The 3rd Affiliated Hospital (The First Hospital of Nanchang), Nanchang University, Nanchang, China; ^2^ The 1st Affiliated Hospital, Jiangxi Medical College, Nanchang University, Nanchang, China

**Keywords:** TRIM proteins, gastric cancer, oncogenesis, targeting strategies, mechanism

## Abstract

Gastric cancer (GC) is a globally prevalent malignant tumor, causing approximately 770,000 deaths in 2020, ranking fourth among all cancers. The tripartite motif (TRIM) protein family is involved in various cellular regulations and has become a key player in the pathogenesis of gastric cancer. This review explores the therapeutic potential of TRIM proteins in gastric cancer, from signaling pathway regulation to precision targeting strategies. Structurally, there are differences in the C-terminal domain of TRIM proteins, which determine their subgroup classification and substrate recognition. Functionally, they regulate multiple signaling pathways that are crucial for the development of gastric cancer. Clinically, many TRIM proteins serve as promising diagnostic and prognostic biomarkers. In terms of therapy, targeting TRIM proteins holds great potential. Strategies include developing small molecule inhibitors targeting specific TRIM domains, such as inhibitors targeting the bromodomain of TRIM24, and exploring PROTAC technology to degrade oncogenic TRIM proteins. Combination immunotherapy targeting TRIM-related pathways may also provide new therapeutic options. However, challenges persist, Including limited understanding of heterotypic polyubiquitination targets/functions of TRIM proteins, insufficient mechanistic/epidemiological insights into their immunomodulatory roles in the tumor microenvironment, underdeveloped TRIM inhibitors for gastric cancer, unevaluated pharmacokinetics/toxicity of inhibitors in preclinical models, and the need to construct complete TRIM biological systems. In summary, TRIM proteins are deeply involved in the biological processes of gastric cancer, and understanding their functions and regulation could lead to the development of more effective precision targeting strategies for gastric cancer treatment.

## 1 Background

Gastric cancer represents a major global health burden in terms of cancer incidence and mortality. Global cancer statistics indicate that in 2020, over one million new cases were recorded, resulting in approximately 769,000 deaths (corresponding to 1 in 13 global cancer-related fatalities), with the disease ranking fourth in mortality worldwide and fifth in incidence (1). The majority of patients present with advanced-stage disease, primarily due to the insidious nature of symptom onset and low rates of regular screening (2). The complex molecular pathogenesis and interpatient heterogeneity contribute to suboptimal prognoses (3). Therefore, uncovering the underlying mechanisms of GC and identifying effective therapeutic targets holds significance.

Comprising over 70 members, the TRIM protein family exhibits a conserved architectural framework, featuring a RING finger domain, one or two B-box zinc-finger motifs, and a coiled-coil helical domain (4). Research has shown that TRIM proteins, functioning as critical modulators of transcriptional regulation, are deeply involved in the oncogenic processes of various malignancies (5). Explorations in various cancer types such as lung (6), liver (7), prostate (8), and breast (9) malignancies have uncovered their multifaceted roles in driving cellular processes critical for oncogenesis. These include modulation of cell proliferation, promotion of metastatic cascades, regulation of metabolic reprogramming, influence on stem cell differentiation, orchestration of the immune microenvironment, and impact on tumor therapeutic responses.

The diverse functionalities of TRIM family proteins, stemming from the structural diversity of their functional domains (7),position them as pivotal regulators in cancer biology. Elucidating their precise molecular mechanisms—particularly in signaling pathway modulation and cellular process orchestration—offers critical opportunities to exploit these proteins as therapeutic targets for gastric cancer. This review systematically synthesizes current knowledge of TRIM proteins’ roles in gastric cancer, dissecting their involvement in oncogenic signaling, metastatic cascades, and therapeutic resistance. By highlighting unresolved mechanistic gaps and emerging precision-targeting strategies—such as pathway-specific inhibitors and immune-modulatory approaches—this work lays groundwork for translating TRIM-focused research into clinical applications, ultimately advancing the development of targeted therapies for this aggressive malignancy.

## 2 Properties of E3 ligase in GC

### 2.1 Summary figure

**Figure 1:**
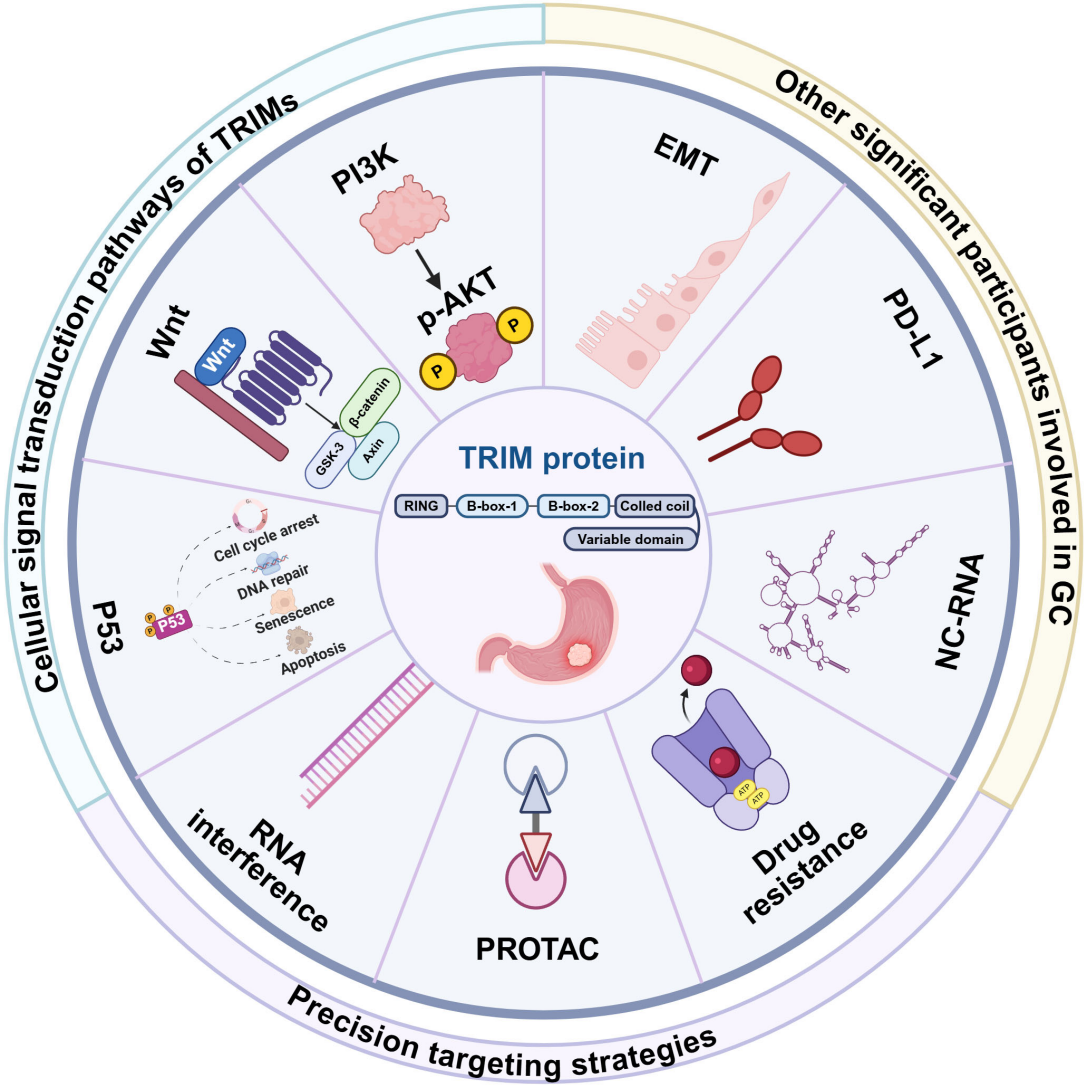
TRIM proteins in gastric cancer: schematic overview of associated mechanisms and research strategies.

**Figure 1** is a schematic diagram summarizing TRIM protein-related mechanisms and research strategies in gastric cancer. Centered on the structure of TRIM proteins, the outer circle presents related content from multiple dimensions: Key participants involved in gastric cancer, including PD-L1, EMT, and ncRNA, which play critical roles in processes such as gastric cancer oncogenesis and progression; TRIM protein-associated signaling pathways, such as the Wnt pathway, P53, and AKT. TRIM proteins can regulate these pathways to influence the biological behaviors of gastric cancer cells; Precision targeting strategies, encompassing PROTAC, RNA interference, and drug resistance, provide potential precise intervention approaches for TRIM protein-targeted and gastric cancer treatment, facilitating the exploration of new directions in gastric cancer diagnosis and therapy.

### 2.2 Structural features of TRIMs

**Figure 2:**
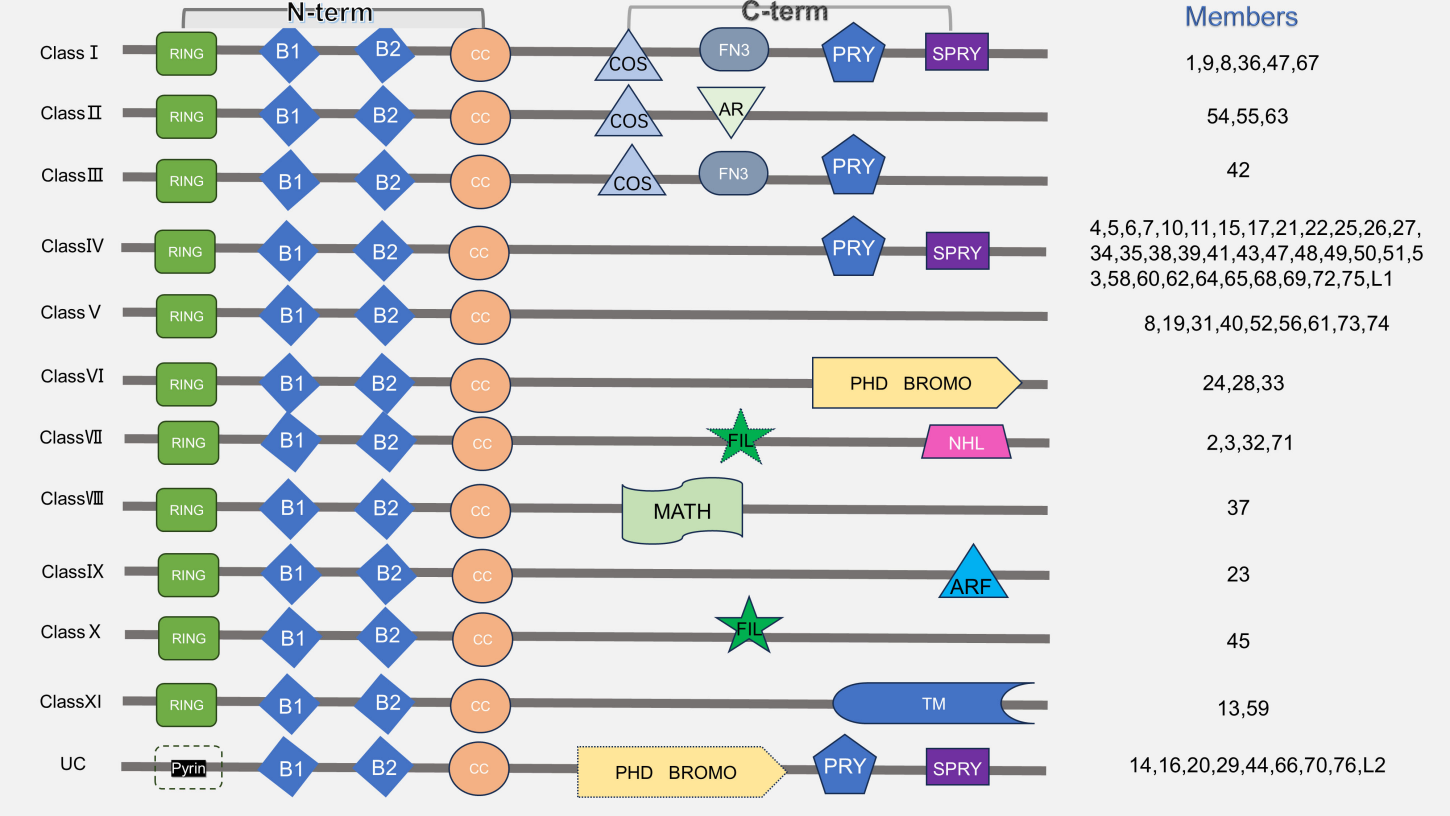
Classification of TRIM family structures. The TRIM family is structurally categorized such that members containing a RING domain are divided into 11 subfamilies (C-I to C-XI), whereas the unclassified (UC) group comprises TRIM proteins lacking this RING domain. Most TRIM genes encode proteins that contain a RING domain, together with B1/B2 domains and a coiled-coil (CC) domain. The C-terminal region encompasses various domains, including the COS (C-terminal subgroup one signature) box, fibronectin type-III (FN3) domain, PRY/SPRY (also referred to as B30.2) domain, acid-rich (ACID) region, plant homeodomain (PHD), bromodomain (BROMO), filamin-type I (FIL) domain, NHL (NCL1, HT2A, and LIN41) domain, MATH (Meprin and TRAF-homology) domain, ADP-ribosylation factor family (ARF) domain, and transmembrane (TM) region.

TRIM proteins, a family of RING finger-containing proteins, predominantly act as E3 ubiquitin ligases (10). Key members like TRIM24, TRIM47, TRIM59, and TRIM50 are classified as E3 ligases owing to their RING domains. Conversely, some TRIM members (e.g.TRIM14, TRIM16, TRIM20) lack zinc finger domains and are thought to lack E3 ligase activity (11). The structure of TRIM proteins is shown in **Figure 2**. Aside from the upregulated and conserved structure at the N-terminus, variable structural differences are observed in the C-terminal domain. TRIM proteins are categorized into 11 subgroups (C-I to C-XI) (12). The PRY/SPRY domain (B30.2), the most prevalent C-terminal motif, mediates target protein/RNA recognition and binding (5). Members of the unclassified (UC) group, devoid of a RING domain, still serve as E3 ligases through their B-Box structure. **Figure 2** elaborates on the structure of TRIM proteins according to different subfamilies, including Class I to XI and UC. The C-terminal domains comprise COS, FN3, PRY/SPRY, PHD, NHL, and ARF domains, among others. Domain-specific functions are detailed in **Table 1**.

**Table 1:** The functions of each domain of TRIMs.

Domain	Core functions	Ref
RING	Catalyzes ubiquitin transfer to target substrates by binding to E2 ubiquitin-conjugating enzymes, essential for ubiquitination-mediated protein degradation or modification.	(13)
B-BOX	B1 subdomain: Exhibits intrinsic E3 ligase activity or enhances RING domain-dependent ubiquitination. B2 subdomain: Collaborates with B1 or RING to modulate substrate recognition and enzymatic efficiency.	(14)
COILED-COIL	Facilitates homodimeric or heteromeric interactions, enabling assembly of multi-protein complexes and subcellular localization critical for signaling pathway activation	(15)
PRY-SPRY	Serves as a protein-RNA interaction hub, mediating recognition of viral components in innate immunity and facilitating substrate binding for ubiquitination-dependent signaling.	(16)
PHD-BROMO	Contains DNA-binding and transcriptional activation properties, acting as a scaffold for protein-protein interactions in chromatin remodeling and gene regulation.	(17)
NHL	Binds specific RNA sequences/structures, regulating mRNA stability and processing, particularly in post-translational control of gene expression.	(18)
MATH	Promotes self-association and hetero-oligomerization, facilitating interactions with TRAF family ubiquitin ligases to modulate inflammatory and immune responses.	(19)
FN3	Functions as a modular scaffold for molecular interactions, often involved in cell surface receptor signaling and extracellular matrix binding.	(20)
ACID	An acidic glutamate-rich region that augments ubiquitin-mediated protein degradation, potentially influencing muscle protein turnover and cellular homeostasis.	(21)
TM (Transmembrane)	Integral to endoplasmic reticulum localization, suppresses inflammatory responses to pathogenic DNA through membrane-associated signaling regulation.	(22)
FIL	Regulates immune system components and TRIM-NHL mRNA stability, linking RNA metabolism to immune response modulation.	(23)
ARF	Participates in intracellular vesicle transport via GTP hydrolysis, influencing membrane trafficking and organelle dynamics.	(24)
COS	Mediates interactions with the microtubule cytoskeleton, contributing to cell polarity and cytoskeletal remodeling during cell migration and division.	(25)
PYRIN	Orchestrates inflammatory and apoptotic pathways, critical for immune surveillance and host defense against pathogens.	(16)

### 2.3 TRIM proteins exhibit oncogenic or suppressive functions in gastric cancer

Recent investigations have underscored the involvement of TRIM proteins in innate immune responses against viral pathogens, attracting considerable scientific scrutiny (26). Emerging evidence reveals that TRIM proteins serve as either oncogenic drivers or suppressive regulators across multiple malignancies, with gastric cancer (GC) as a paradigmatic case. For example, downregulated TRIM3 expression was linked to inhibited proliferation and migration in GC cells, with favorable prognostic significance attributed to its suppression of β-catenin and cyclin D expression (27). Conversely, overexpression of TRIM11 in GC tissues correlated with enhanced cell invasion and EMT through Axin1 destabilization in the Wnt/β-catenin pathway, predicting poor patient outcomes (28). TRIM14 overactivity promoted metastatic potential via AKT/mTOR signaling, with its upregulation associated with advanced clinical stages and chemoresistance (29, 30). In contrast, TRIM58 exerted suppressive effects by ubiquitinating β-catenin to inactivate its signaling, with lower expression levels correlating with accelerated cell cycle progression (31). TRIM59, by inhibiting the p53 pathway, promoted clone formation and migration, with its overexpression emerging as an unfavorable prognostic marker (32).

These diverse roles highlight TRIM proteins as central nodes in GC pathogenesis, with their functions mediated through intricate signaling networks—from Wnt/β-catenin dysregulation to metabolic reprogramming and immune evasion. **Table 2** focuses on TRIM proteins in gastric cancer, summarizing their subfamily classification, expression patterns in gastric cancer, functional roles, prognostic relevance, as well as the native or control biological tissues, tested cell lines, specific biological effects, molecular mechanisms, potential therapeutic strategies, and related references involved in the research. It comprehensively presents the multi-dimensional characteristics of TRIM proteins in gastric cancer and their research foundations.

**Table 2:** Structure, expression patterns, functional roles, mechanisms, and possible treatment strategies of TRIM proteins in gastric cancer.

Gene	Subfamily	Expression	Role	Prognostic relevance	Tissues (Native/Control)	Tested cells	Effect	Molecular mechanisms	Possible treatment strategies	Refs
TRIM3	Class VII	Low	Suppressive	Favorable	20/20	SGC-7901, MGC-803 (GC); GES-1 (normal)	Growth inhibition; migration, EMT suppression	Inhibits β-catenin and cyclin D gene expression	-	(27)
TRIM11	Class IV	High	Oncogenic	Unfavorable	36/36	GES-1 (normal); AGS, HGC-27, SGC-7901, MGC-803 (GC)	Proliferation, migration, invasion, EMT promotion	Wnt/β-catenin pathway activation	PROTACs	(33)
TRIM3	Class VII	High	Oncogenic	Unfavorable	40/40	NA	Invasion depth, tumor size, lymph node metastasis association	NA	Exosomes; PROTACs	(34)
TRIM7	Class IV	Low	Suppressive	Favorable	80/NA	GES-1 (normal); AGS, HGC-27 (GC)	Migration, invasion, metastasis suppression; Synergistic Temozolomide anti-tumor	Targeting SLC7A11-mediated ferroptosis induction	–	(35)
TRIM11	Class IV	High	Oncogenic	Unfavorable	150/150	GES-1 (normal); HGC-27, SGC-7901, BGC-823, MKN45, MGC-803 (GC)	Proliferation, migration promotion	Axin1 destabilization to activate Wnt/β-catenin pathway	PROTACs	(28)
TRIM14	UC	High	Oncogenic	Unfavorable	117/117	GES-1 (normal); BGC-823, MKN45, AGS, SGC-7901, MGC-803 (GC)	Migration, invasion, metastasis, EMT, chemoresistance promotion	AKT/mTOR pathway activation	RNA interference; PROTACs	(29, 30)
TRIM15	Class IV	Low	Suppressive	Favorable	134/134	AGS, MKN-1 (GC)	Invasion suppression	NA	-	(36)
TRIM15	Class IV	High	Oncogenic	Unfavorable	275/275	HGC-27, MGC-803 (GC)	Invasion depth, tumor size, lymph node metastasis association	NA	PROTACs	
TRIM16	UC	Low	Suppressive	NA	40/40	NA	NA	β-catenin, cyclin D, and BCL2 gene accumulation	-	(37)
TRIM16	UC	High	Oncogenic	NA	10/10	GES-1 (normal); NCI-N87, MKN-28, BGC-823, AGS, SGC-7901, HGC-27 (GC)	Invasion, migration promotion	NA	RNA interference; PROTACs	(38)
TRIM17	Class IV	High	Oncogenic	NA	82/82	AGS, HGC-27, MKN45, NCI-N87 and HEK293T	proliferation and survival; increased chemotherapy drug sensitivity	BAX ubiquitination and proteasomal degradation	PROTACs	(39)
TRIM21	Class IV	Low	Suppressive	Favorable	64/64	BGC-823, SGC-7901 (GC)	Proliferation inhibition, apoptosis induction, improve gastric cancer apatinib therapy	STAT1 degradation; EZH1 protein suppression	-	(40, 41)
TRIM22	Class IV	Low	Suppressive	Favorable	90/90	GES-1 (normal); 746T, AGS, HGC-27, MGC-803 (GC)	Proliferation, colony formation, migration inhibition	Smad2 protein interaction and TGF-β pathway inhibition	–	(42)
TRIM23	Class IX	High	Oncogenic	Unfavorable	81/40	GES-1 (normal); MKN45, AGS, SGC-7901, HGC-27, BGC-823, MGC-803 (GC)	Tumor size, depth of invasion, differentiation, lymph node metastasis association	NF-κB signaling pathway activation	PROTACs	(43)
TRIM24	Class VI	High	Oncogenic	Unfavorable	133/20	BGC-823, AGS, SGC-7901, MKN-1, HGC-27 (GC)	Proliferation promotion	AKT pathway activation	RNA interference; PROTACs	(44)
TRIM24	Class VI	High	Oncogenic	Unfavorable	90/60	GES-1 (normal); MGC-803, HGC-27, AGS, BGC-823, SGC-7901 (GC)	Proliferation, migration, invasion, apoptosis, metastasis, cell cycle regulation	Wnt/β-catenin pathway activation		(45)
TRIM24	Class VI	High	Oncogenic	Unfavorable	12/12	GES-1 (normal); SGC-7901, AGS, MGC-803, BGC-823, HGC-27 (GC)	Proliferation promotion	PI3K/AKT and Wnt/β-catenin pathways activation		(46)
TRIM25	Class IV	Low	Suppressive	Favorable	90/82	MGC-803, BGC-823, SGC-7901 (GC)	NA	NA	-	(47)
TRIM27	Class IV	High	Oncogenic	Unfavorable	92/92	GES-1 (normal); AGS, BGC-823, MGC-803, HGC-27, SGC-7901, MKN45 (GC)	Proliferation promotion,;inducing 5-FU sensitivity	Hippo-BIRC5 pathway activation;	PROTACs;	(48)
TRIM29	UC	High	Oncogenic	NA	NA/NA	GES-1 (normal); BGC-823, MGC-803 (GC)	Proliferation, cell cycle, apoptosis regulation	Wnt/β-catenin signaling pathway activation	RNA interference; PD-L1 inhibitors; PROTACs	(49)
TRIM29	UC	High	Oncogenic	Unfavorable	124/124	NA	NA	NA		(50)
TRIM31	Class V	High	Oncogenic	NA	170	GES-1 (normal); SGC-7901, AGS, MGC-803, MKN45, HGC-27 (GC)	Colony formation, proliferation, invasion promotion	Axin1 protein stability regulation to activate Wnt/β-catenin pathway	PROTACs	(51, 52)
TRIM32	Class VII	High	Oncogenic	Unfavorable	142/NA	MKN45, MKN74 (GC)	Proliferation, apoptosis regulation	NA	PROTACs	(53)
TRIM32	Class VII	High	Oncogenic	Unfavorable	81/20	GES-1 (normal); SGC-7901, MKN-28, AGS, BGC-823 (GC)	Proliferation, migration, invasion, colony formation promotion	β-catenin signaling pathway activation		(54)
TRIM32	Class VII	High	Oncogenic	Unfavorable	876/NA	GES-1 (normal); MKN45, AGS, HGC-27, NCI-N87, MKN74 (GC)	Proliferation, apoptosis regulation	Akt signaling pathway activation		(55)
TRIM37	Class VIII	High	Oncogenic	Unfavorable	124/124	Kato-III, NUGC4, HGC-27, MKN7, MKN28, MKN45, MKN74 (GC)	Apoptosis inhibition, tumor growth, invasion, metastasis promotion; cisplatin resistance	SIP1-mediated EMT induction	RNA interference; PROTACs	(56, 57)
TRIM40	Class V	Low	Oncogenic	NA	NA/NA	HEK293T, HeLa, SW480, IEC-6 cells (non-GC)	NA	NF-κB signaling pathway activation	–	(58)
TRIM44	UC	High	Oncogenic	Unfavorable	112/7	KatoIII, NUGC4, MKN74, MKN28, MKN45, AGS, HGC-27 (GC)	Proliferation, migration, invasion promotion	NA	PROTACs	(59)
TRIM44	UC	High	Oncogenic	Unfavorable	75/NA	NA	Chemotherapy response regulation	TRIM44/14-3-3ζ/β-catenin signaling pathway		(60)
TRIM47	Class IV	High	Oncogenic	Unfavorable	136/30	AGS (GC)	Apoptosis inhibition, EMT promotion	NF-κB signaling pathway activation	PROTACs	(61)
TRIM50	Class IV	Low	Suppressive	NA	415/34	GES-1 (normal); MKN-45, SGC-7901, AGS, MGC-803, BGC-823, HGC-27, MKN-28 (GC)	Proliferation, cell cycle, migration, invasion inhibition	Wnt/β-catenin signaling pathway inhibition	–	(62)
TRIM54	Class II	High	Oncogenic	Unfavorable	4/4	GES-1 (normal); AGS, MGC-803, HGC-27 (GC)	Proliferation, migration, invasion, metastasis promotion	NA	-	(63)
TRIM58	Class IV	Low	Suppressive	Favorable	23/23	GES-1 (normal); MKN45, HGC-27, AGS,BGC-823, (GC)	Proliferation, cell cycle inhibition	β-catenin signaling pathway inactivation	–	(31)
TRIM59	Class XI	High	Oncogenic	Unfavorable	156/122	GES-1 (normal); SGC-7901, MKN45, AGS, BGC-823, N87, SNU1, SNU5 (GC)	Proliferation, clone formation, migration promotion	p53 signaling pathway activation	PROTACs	(32)
TRIM65	Class IV	High	Oncogenic	NA	NA/NA	GES-1 (normal); SNU1, AGS, NCI-N87, HGC-27 (GC)	Proliferation, invasiveness, migration promotion	PPM1A ubiquitination degradation upregulation	PROTACs	(64)
TRIM69	Class IV	Low	Suppressive	Favorable	162/162	NA	Resistance inhibition, metastasis suppression; suppressed the anoikis resistance	PRKCD degradation via ubiquitin-proteasome pathway	-	(65)

## 3 Mechanisms underlying TRIM proteins in GC

### 3.1 Cellular signal transduction pathways of TRIMs

#### 3.1.1 Wnt/β-catenin pathway

The Wnt/β-catenin signaling cascade acts as a central modulator in gastric tumorigenesis (66). This pathway, known as the canonical Wnt pathway and conserved throughout evolution, regulates vital physiological functions such as cell differentiation, apoptosis, invasion, and maintaining tissue homeostasis (67). Activation of the Wnt/β-catenin axis prompts a rapid increase in cytoplasmic β-catenin levels. This protein then translocates to the nucleus, where it upregulates the transcription of cyclin D1 and c-Myc, thereby driving cell proliferation and metastatic spread (67, 68). In contrast, inhibition of Wnt signaling leads to the phosphorylation of accumulated β-catenin by the APC-Axin1/2-GSK-3β complex, effectively dampening its oncogenic activity (68).

TRIM31 physically associates with Axin1, triggering its ubiquitin-mediated proteasomal degradation. This Axin1 depletion activates the Wnt/β-catenin signaling axis, accelerating gastric cancer (GC) initiation and progression (51). Additionally, TRIM32 propels GC cell proliferation, motility, and invasive capacity by activating the β‐catenin signal pathway (54).

Upregulated TRIM11 in GC cells elevates β-catenin, c-Myc, CyclinD1, and vimentin, potently enhancing cell proliferation, migration, and invasiveness (33). Molecular assays (Western blotting, immunofluorescence) demonstrated that TRIM11 overexpression facilitates β-catenin nuclear shuttling, a pivotal step in pathway activation (33). In GC tissues, TRIM11 expression inversely correlates with Axin1 protein levels (28), underscoring its role in destabilizing Axin1 and activating Wnt/β-catenin signaling to drive tumor progression (28).

**Figure 3:**
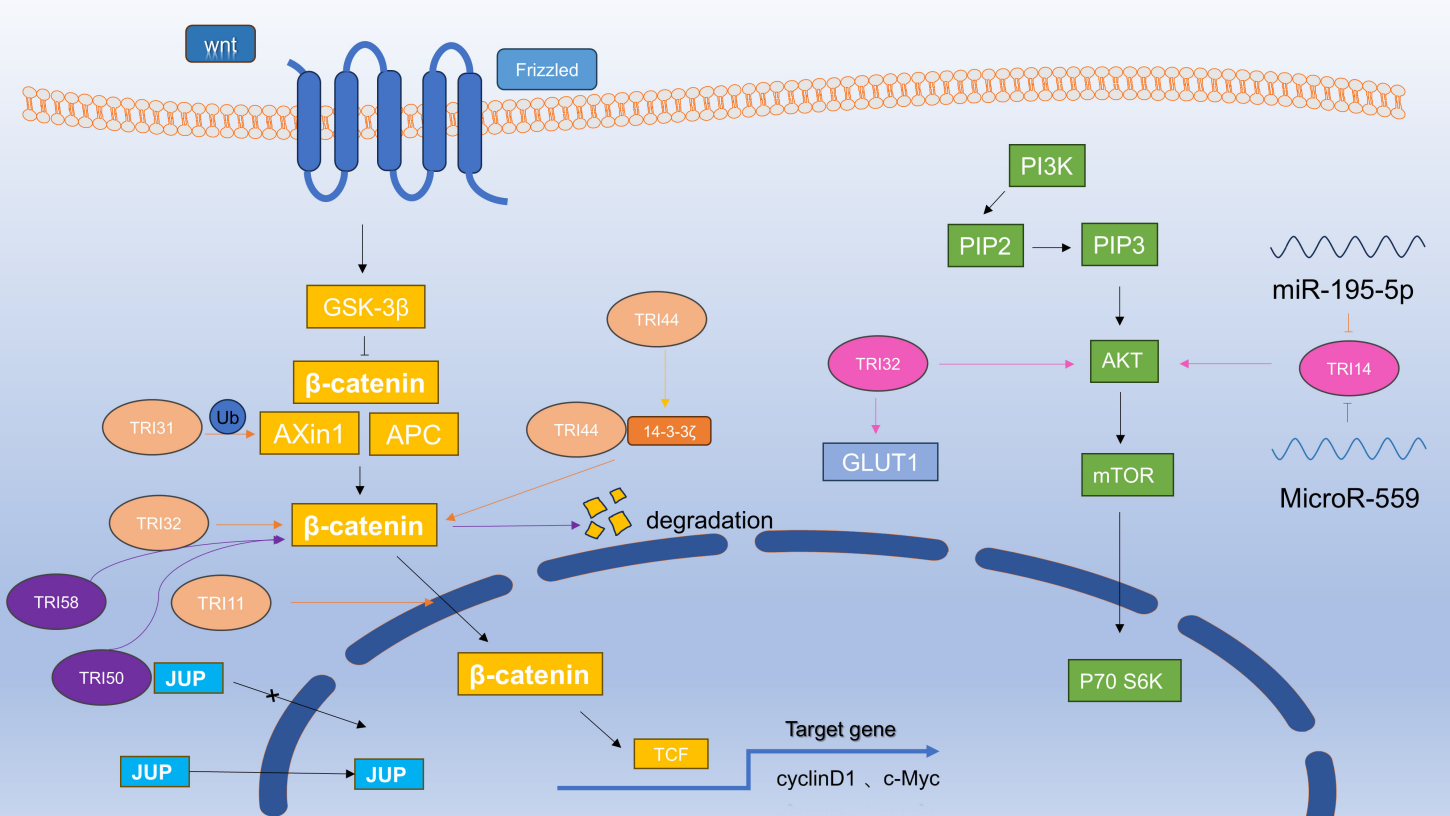
Relationship of TRIM proteins with the Wnt/β-catenin and PI3K/AKT pathways in gastric cancer.

In GC patients, β-catenin expression positively correlates with TRIM44 and TRIM24 (45, 69). TRIM44 drives oncogenic processes in GC cells, partially via β-catenin/Wnt signaling. In **Figure 3**, TRIM44 stabilizes 14-3-3ζ: it binds 14-3-3ζ via its B-box domain and deubiquitinates it using the zinc finger domain, preventing proteasomal degradation (60). This 14-3-3ζ accumulation upregulates β-catenin, fueling gastric cancer hematopoietic stem cell proliferation, tumorigenesis, and chemoresistance (60). TRIM52 modulates the Wnt/β-catenin pathway to enhance GC cell proliferation, invasion, and migration (70). Meanwhile, TRIM29 overexpression in GC patients associates with poor prognosis, potentially via the β-catenin/Cyclin D/Bcl2 axis, positioning it as a candidate independent prognostic marker (49).

TRIM50, a recently discovered member of the TRIM protein family (71), functions as a negative regulator of gastric tumorigenesis. In contrast to oncogenic TRIM proteins,overexpressed TRIM50 exerts suppressive effects on GC cell proliferation and metastatic potential (62). As illustrated in **Figure 3**, the JUP protein, a paralog of β-catenin, can also interact with TRIM50, However, when bound to the JUP protein, TRIM50 cannot enter the nucleus. Another study investigated TRIM58 overexpression in nude mice, demonstrating a notable decrease in tumor growth and weight, along with an elevation in tumor cell apoptosis. In addition, the results indicated a decrease in β-catenin expression, suggesting that TRIM58 inhibits gastric cancer tumor growth by ubiquitination, inactivating β-catenin signaling (31). Decreased TRIM16 expression also led to the accumulation of β-catenin and ultimately exerted a tumor suppressive effect (37). The complex regulatory interactions between TRIM family members and the Wnt/β-catenin signaling cascade are visualized in **Figure 3**, depicting their bidirectional molecular crosstalk. Although further detailed studies are required, these TRIM proteins are promising targets for GC therapy.

#### 3.1.2 PI3K/AKT

The PI3K/AKT/mTOR signaling cascade represents a fundamental pathway implicated in essential cellular processes (72). Its dysregulated activation governs key mechanisms in multiple human malignancies, including gastric cancer, such as autophagy, epithelial-mesenchymal transition (EMT), apoptosis, chemoresistance, and metastatic progression (72, 73).

A previous study reported that overexpression of TRIM14 promotes p-AKT, p-mTOR, and p-P70S6K expression, while TRIM14 knockout induces opposite effects; furthermore, AKT inhibition has been demonstrated to counteract the pro-migratory and pro-invasive effects of TRIM14 in gastric cancer cells (30). This suggests that the AKT pathway at least partially mediates the effects of TRIM14 on GC migration and invasion (30). Additionally, enhancement of the activity of AKT increases chemoresistance in GC (74). These are in **Figure 3**. TRIM14 facilitates gastric cancer cell migration and invasion by modulating epithelial-to-mesenchymal transition (EMT) through AKT signaling activation, a process regulated by miR-195-5p (30). Additionally, microRNA-559 inhibits gastric cancer progression by activating the AKT signaling pathway via targeting TRIM14 (75).

TRIM32 overexpression significantly inhibits apoptosis in GC cells, which can be reversed by AKT inhibitors (54). Interestingly, silencing of TRIM32 remarkably suppressed AKT phosphorylation in a time-dependent fashion (54). Moreover, inhibition of TRIM32 was also found to inhibit the glycolysis-related protein glucose transporter 1 (GLUT1), a key mediator of glucose uptake in GC cells (54). Collectively, these findings suggest that TRIM32 may drive gastric cancer cell proliferation by enhancing AKT activation and glucose transport (54). Although Akt inhibitors cannot prevent the effects of TRIM24 on proliferation, Akt inhibitors significantly block the effect of TRIM24 on chemoresistance, and inhibition of Akt could counteract the chemoresistance-enhancing effect of TRIM24 (44). The relationship between the TRIM proteins and the PI3K/AKT signaling cascade is illustrated in **Figure 3**.

#### 3.1.3 P53

The well-characterized tumor suppressor p53 safeguards genomic integrity in a context-dependent manner (76). The RING domain of TRIM59 is essential for its interaction with the p53 protein complex, which in turn modulates p53 ubiquitination. Studies have shown that TRIM59 overexpression in gastric cancer suppresses the canonical p53 ubiquitin E3 ligase activity, a mechanism identified as central to TRIM59-mediated p53 regulation (32). Murine bi-minute-2 (MDM2) is a classic P53 ubiquitin E3 ligase, which was found to contribute to the regulation of P53 by TRIM59 (32).

Surprisingly, inhibition of P53’s nuclear activity by TRIM29 enhanced cancer cell proliferation (77). In contrast to TRIM59, TRIM29 does not possess a RING finger domain and inhibits p53 by blocking its nuclear translocation (77). Moreover, prior investigations have revealed that the interaction between TRIM29 and HDAC9 disrupts the binding affinity and deacetylation capacity of TRIM29, thus influencing the interaction between TRIM29 and p53 (78).

### 3.2 Other significant participants involved in GC conditioning along with TRIMs

#### 3.2.1 EMT

Epithelial-mesenchymal transition (EMT) is a dynamic process that enables epithelial cells to acquire characteristics of mesenchymal cells (79). This biological process is frequently activated to drive cancer progression, metastatic dissemination, and the development of therapeutic resistance. Depending on the signaling pathways involved, TRIMs can either promote or hinder EMT, regulating cancer progression and treatment resistance.

An increase in TRIM14 expression significantly lowers the levels of the epithelial cell marker E-cadherin, whereas it raises the concentrations of mesenchymal markers such as N-cadherin and vimentin (30). Conversely, TRIM14 depletion results in the opposite phenotypic shift (30). These findings suggest that TRIM14 is an activator of the EMT process in GC, which regulates the transition of epithelial cells to the mesenchymal phenotype by activating AKT signaling, thereby promoting the migration and invasion of gastric cancer (30).

Furthermore, overexpression of TRIM37 leads to the upregulation of SIP1 expression, which in turn accelerates the progression of EMT, thereby enhancing the migration and invasion capacity of GC cells (56). SIP1 short hairpin RNA (shSIP1) significantly reversed TRIM37-induced EMT and partially reversed TRIM37-promoted cell invasion (56). These results implied that TRIM37 prompted GC cell invasion and EMT through the activation of the transcription factor SIP1 (56). However, transcription factors usually regulate genes that express and function cooperatively on the target, and whether the EMT transcription program triggers a transcription factor coordination response requires further research. Certain TRIM proteins, such as TRIM11, indirectly control the process of EMT by operating within signal transduction routes like the WNT/β-catenin pathway (33).

#### 3.2.2 PD-L1

PD-L1, the third member of the B7 family, binds to PD-1 to trigger apoptosis, leading to functional impairment and depletion of T cells. This process suppresses the activation, proliferation, and anti-tumor activity of tumor antigen-specific CD8+ T cells, thereby facilitating tumor immune escape (80, 81).

**Figure 4:**
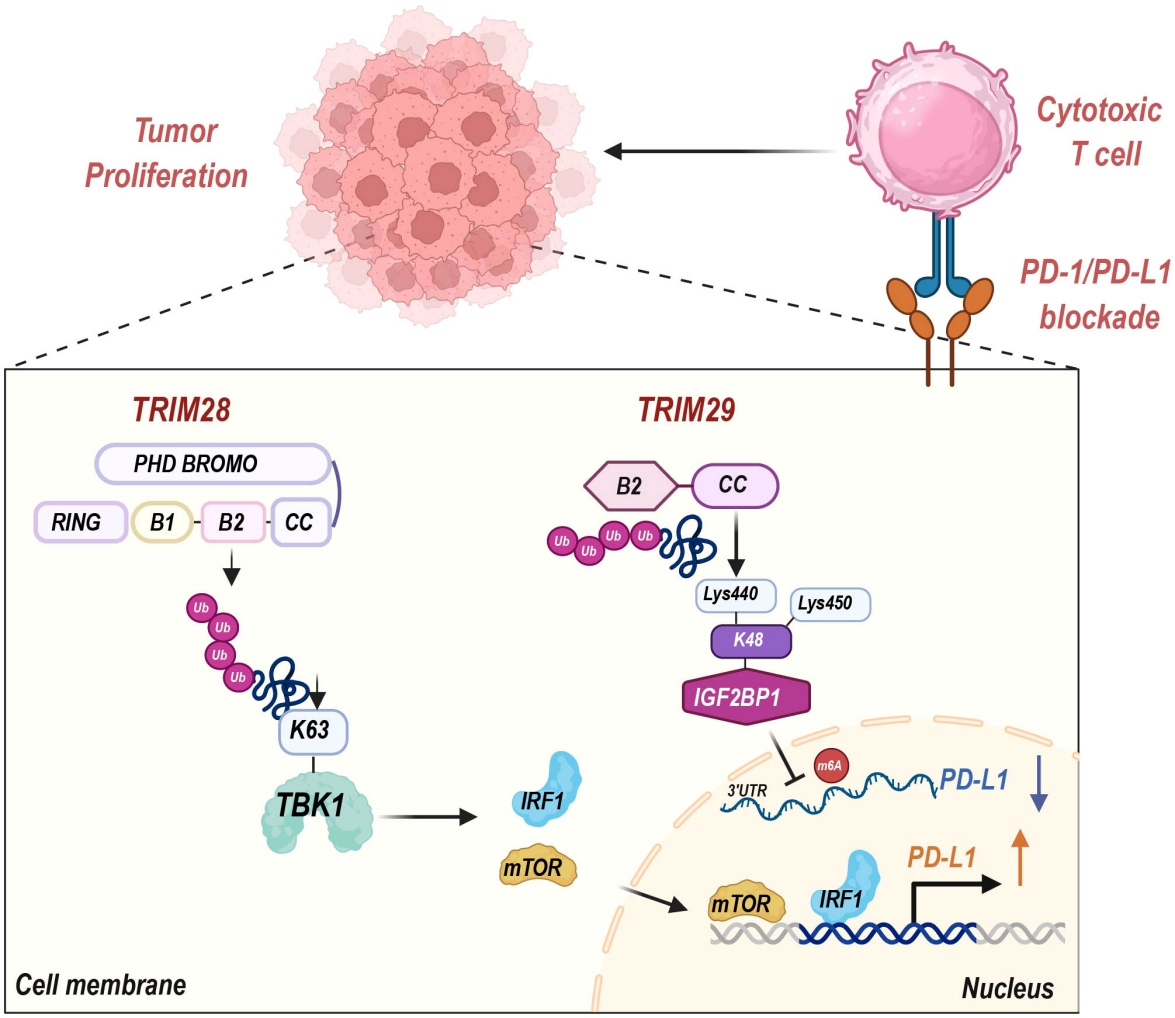
Involvement of TRIM proteins in the immune evasion of gastric cancer cells.

As shown in **Figure 4**, TRIM28 is pivotal in maintaining gastric cancer stem cell viability and immune regulation (82). It directly interacts with PD-L1, stabilizing the protein through inhibiting its ubiquitination and promoting SUMOylation. The B-box2 domain of TRIM28 is essential for this interaction, while the C-terminal cytoplasmic domain of PD-L1 is critical for binding to TRIM28 (83). Additionally, reducing PD-L1 expression attenuates TRIM28-induced tumor growth by enhancing CD8+ T cell infiltration (83). Previous research revealed that TRIM28 promotes the polyubiquitination of K63 of Tbk1, activating both the TBK1-IRF1 and TBK1-mTOR signaling pathways. This mechanism increases PD-L1 abundance and enables gastric cancer cells to evade immune surveillance (83).

However, recent *in vivo* and *in vitro* investigations demonstrate that TRIM29 enhances anti-tumor T cell immunity via the IGF2BP1/PD-L1 axis in gastric cancer (84). As shown in **Figure 4**, IGF2BP1 is the TRIM29 interaction element, which promotes the stabilization and expression of PD-L1 mRNA in a 3’ non-coding region in an m6A-dependent manner (84). TRIM29 interacts with IGF2BP1 and induces K48-linked ubiquitination of IGF2BP1 at Lys440 and Lys450 residues, leading to proteasomal degradation. Consequently, PD-L1 down-regulation enhances anti-tumor immunity in gastric cancer (84).

Most PD-L1 inhibitors are monoclonal antibodies, such as Atezolizumab. These antibodies target PD-L1 through the specific binding capacity of monoclonal antibodies, thereby exerting antitumor immune effects. In gastric cancer with high TRIM28 expression: TRIM28 stabilizes PD-L1 protein by inhibiting PD-L1 ubiquitination and promoting its SUMOylation; meanwhile, it increases PD-L1 abundance by activating the TBK1 pathway, exacerbating immune evasion. Under such circumstances, PD-L1 inhibitors can directly block PD-L1/PD-1 binding, further reduce PD-L1 expression, enhance therapeutic efficacy, and mitigate immune resistance. TRIM29 downregulates PD-L1 by degrading IGF2BP1, thereby enhancing antitumor immunity. If TRIM29 is functionally deficient, the accumulation of IGF2BP1 will lead to high PD-L1 expression, and PD-L1 inhibitors can then effectively block the abnormally elevated PD-L1. The interaction mechanism between TRIM proteins and PD-L1 provides a new idea for the development of clinical immunotherapy against gastric cancer, Upregulated lighting the translational potential of targeting these axes to overcome immune resistance.

#### 3.2.3 Noncoding RNA

In addition to the protein-coding genes implicated in gastric carcinogenesis, numerous non-coding RNAs (ncRNAs) have also been linked to tumor proliferation. The diagnostic and therapeutic roles of ncRNAs are further influenced by the interaction with TRIM proteins.

Long non-coding RNA (lncRNA) ASB16 antisense RNA 1 (ASB16-AS1) is identified as an oncogenic factor across multiple cancer types (85, 86), enhances TRIM37 expression in GC cells through interaction with miR-3918 and miR-4676-3p (87). This interaction initiates the activation of the NF-κB pathway, thereby promoting gastric cancer cell proliferation, apoptotic resistance, and cisplatin tolerance (87). As previously discussed, TRIM14 drives gastric cancer cell migration and invasion via AKT signaling activation, with its activity under the regulation of miR-195-5p (30). Conversely, MicroRNA-559 suppresses gastric cancer progression by promoting AKT signaling activation through TRIM14 targeting (75).

A validated functional target of miR-511 is TRIM24 (TIF1α). Endogenous miR-511 inhibition results in enhanced GC cell growth, colony formation ability, and accelerated cell cycle progression (46). miR-511 exerts its antitumor effects by inactivating both the PI3K/AKT and Wnt/β-catenin pathways via TRIM24 suppression (46). Additionally, TRIM29 functions as an oncogenic protein in gastric cancer, with its expression regulated by miR-185 (49). The oncogenic effects of lncRNA ELFN1-AS1are mediated through acting as a molecular sponge for miR-211-3p, consequently elevating the expression of TRIM29, the identified target gene of miR-211-3p (88, 89).

A prior investigation uncovered that a long noncoding RNA specifically associated with gastric cancer distant metastasis correlates with TRIM16 expression, which has been demonstrated to promote tumor cell invasiveness *in vitro (* 38). The regulatory relationships between TRIM proteins and noncoding RNAs are tabulated in **Table 3**. These findings underscore the therapeutic potential of ncRNA-TRIM axis targeting, including RNA interference, small-molecule inhibition, and gene editing strategies. By leveraging the interactions between lncRNAs/miRNAs and TRIM proteins, these approaches may overcome tumor resistance and provide precision therapies for gastric cancer, with promising opportunities for combination with chemotherapy or immunotherapy.

**Table 3:** TRIM proteins are the targets of non-coding RNAs.

TRIM	Upstream ncRNA	Associated molecules or mechanisms	Refs
TRIM37	miR-3918 miR-4676-3p	NF-κB pathway	(87)
TRIM14	miR-195-5p	AKT signaling	(30)
	MicroRNA-559	AKT signaling	(75)
TRIM24(TIF1α)	miR-511	The PI3K/AKT and Wnt/β-catenin pathways	(46)
TRIM29	miR-185, miR-211-3p	–	(49, 88)
TRIM16	LncRNA	–	(38)

#### 3.2.4 Other proteins and signaling pathways

Knockdown of TRIM65 inhibits gastric cancer cell growth and aggressiveness by inhibiting PPM1A ubiquitination and blocking TBK1 phosphorylation (64). TRIM54 promotes GC cell proliferation, migration, and invasion via the TRIM54/FLNC axis, which enhances K63-linked ubiquitination of filamin C (FLNC) (63).

TRIM22 directly interacts with Smad2 to suppress gastric cancer cell proliferation, and Smad2 overexpression counteracts TRIM22-mediated inhibition of cell proliferation and migration (42). Lysyl oxidase-like 2 (LOXL2), a matrix enzyme expressed in hepatic fibrotic regions, facilitates fibrous collagen I cross-linking (90). TRIM44 directly binds to LOXL2 and regulates its stability to remodel the tumor extracellular matrix and influence tumor immunity (91).

## 4 Overcoming chemoresistance via signaling pathway disruption

Temozolomide (TMZ) is an oral alkylating antineoplastic agent whose antitumor activity is primarily achieved through the rapid spontaneous degradation *in vivo* to generate the active metabolite5-(3-methyltriazene-1-yl)imidazole-4-carboxamide (MTIC)2. Studies have shown that TRIM7 can synergistically act with temozolomide against ferroptosis (92). Ferroptosis represents a regulated type of cellular demise triggered by prolonged lipid peroxidation stress (93). The upregulation of solute carrier family 7 member 11 (SLC7A11), a cystine glutamate transporter, partially facilitates tumor progression by suppressing ferroptosis (94). Recent investigations have revealed that E3 ubiquitin ligase TRIM7 inhibits gastric cancer development via targeting SLC7A11 (35). Mechanistically, The SPRY domain of TRIM7 engages in direct interaction with SLC7A11 to promote the polyubiquitination SLC7A11 of Lys48 linkage, thereby inhibiting the SLC7A11/GPX4 axis (35). Although temozolomide combined with TRIM7 could theoretically exert a synergistic anti-tumor effect by enhancing ferroptosis, as of now, this combination therapy has not been studied clinically.

Knockdown of TRIM17 enhances drug-induced apoptotic responses and significantly reduces the IC50 values of chemotherapeutic drugs (such as ABT-263 and oxaliplatin), thereby indicating that cells become more sensitive to the drugs (39). TRIM17 was inversely associated with BAX expression levels in gastric cancer cells. Functioning as an E3 ubiquitin ligase, TRIM17 facilitates BAX ubiquitination and subsequent proteasomal degradation (39). BAX-dependent apoptotic deletion in the presence of apoptosis stimuli, thereby promoting tumor cell survival and chemotherapeutic tolerance (39, 95). In addition, TRIM17 acts as a negative regulator of the apoptotic reaction and is triggered by cancer treatments; Knockdown of TRIM17 was observed to improve the efficacy of chemotherapy, demonstrating therapeutic potential in gastric cancer (39).

Apatinib is an oral-administered vascular endothelial growth factor receptor 2 (VEGFR-2) tyrosine kinase inhibitor (TKI)-based anti-cancer agent, whose antitumor activity is primarily achieved through inhibiting VEGFR-2-mediated angiogenesis to block tumor blood supply and induce tumor cell apoptosis (96). TRIM21 overexpression significantly enhances apoptosis and inhibits stem cell properties in GC cells (40); moreover, TRIM21 can inhibit EZH1 (an enhancer of zeste homolog 1) stability to improve gastric cancer apatinib therapy (41). STAT1 (Signal transducer and activator of transcription 1) represses the transcriptional activity of TRIM21 in gastric cancer (40). TRIM21 additionally regulates the ubiquitination of STAT1 in a GTP-dependent manner, serving as the ubiquitin E3 ligase for STAT1 in GC (97). Moreover, cytoplasmic TGM2 (transglutaminase 2) facilitates the dissociation of TRIM21 from STAT1 and preserves STAT1 stability. Functioning as a GTP-binding enzyme, TGM2 promotes gastric cancer progression (97).

5-Fluorouracil (5-FU) is an antimetabolite-class antineoplastic drug, whose antitumor activity is primarily achieved through conversion to active metabolites that inhibit thymidylate synthase, block deoxythymidylate synthesis, and disrupt DNA replication and RNA transcription (98). In the MGC-803 and HGC-27 gastric cancer cell lines, TRIM27 downregulation exerts its effect by first upregulating large tumor suppressor kinase 2 (LATS2), which in turn leads to the inhibition of Yes-associated protein 1 (YAP1), ultimately resulting in the suppression of BIRC5 (48). Down-regulation of TRIM27 can inhibit cell proliferation by inhibiting the Hippo-BIRC5 pathway and inducing 5-FU sensitivity in gastric cancer (48).

PRKCD, as a member of the PKC (protein kinase C) family of serine/threonine-specific kinases, interacts with p53 to modulate cisplatin-induced, caspase-3-mediated apoptosis in gastric cancer (99).TRIM69 suppresses drug resistance and metastatic progression in gastric cancer via the TRIM69-PRKCD/BDNF regulatory axis (65). Through its B-box domain, TRIM69 engages in direct interaction with PRKCD, mediating K48-linked polyubiquitination of this protein. This process promotes PRKCD ubiquitination and subsequent proteasomal degradation, thereby reducing PRKCD stability. Concurrently, TRIM69 inhibits brain-derived neurotrophic factor (BDNF) production in a PRKCD-dependent fashion. Collectively, these mechanisms result in diminished drug resistance and suppressed metastasis in GC cells (65).

Cisplatin (cis-DDP, CDDP) is a platinum-based metal complex antineoplastic agent (100), whose antitumor activity is primarily achieved through forming intra- and interstrand crosslinks with DNA, inducing DNA damage responses, and triggering apoptosis or cell cycle arrest.TRIM37 is involved in promoting malignant progression and cisplatin (CDDP) resistance in gastric cancer, and correlates with tumor recurrence in GC patients after gastrectomy (57).

## 5 Diagnostic/prognostic potential of TRIM proteins in gastric cancer

As previously discussed, numerous TRIM proteins serve as critical regulators of key signaling pathways in gastric cancer. Patients with tumors displaying elevated TRIM32 expression were observed to have notably reduced postoperative overall survival and relapse-free survival compared to those with low TRIM32 expression (53). Moreover, tumors with upregulated TRIM32 levels were linked to an enhanced risk of postoperative recurrence, particularly hematogenous recurrence (53).

A higher expression of TRIM15 (101), TRIM23 (43), and TRIM47 (61) was detected in gastric cancer tissues relative to normal tissues. This heightened expression demonstrated a significant association with tumor invasion depth, lymph node metastasis, advanced TNM stage, and shortened overall survival (OS). Notably, TRIM15 exhibited abundant expression in normal gastric mucosa when compared with tumor tissues. Reduced TRIM15 levels in gastric adenocarcinoma specimens were closely linked to worse patient prognosis (36). Another study reported that TRIM36 may be an important factor influencing the clinical prognosis of GC patients undergoing radiotherapy (102). High TRIM36 expression correlated with enhanced radiosensitivity in gastric cancer, with higher OS rates noted among irradiated patients with elevated TRIM36 levels (102).

TRIM37 is closely linked to clinical metastasis and adverse prognosis in GC through triggering SIP1-mediated EMT (56). This protein regulates the impact of lncRNA ASB16-AS1 on GC cell proliferation, stemness, chemoresistance, and tumorigenesis (87). Clinically, TRIM37 expression correlates with male gender, poor tissue differentiation, venous/lymphatic invasion, advanced PT/PN stages, and increased recurrence risk. These findings underscore its value as a prognostic biomarker and potential malignant phenotype determinant in GC.

Early studies identified TRIM29 as a molecular marker for lymph node metastasis in gastric cancer (50). Determining TRIM29 expression in preoperative gastroscopic biopsy tissue is of great value for predicting lymph node metastasis of gastric cancer before surgery (103). TRIM29 also demonstrated prognostic significance in postoperatively resected gastric cancer patients, with its expression identified as an independent prognostic indicator for overall survival (104). High TRIM44 expression was closely correlated with advanced macroscopic features, lymphatic invasion, elevated GC recurrence rate, and poorer overall survival compared to non-expressing tumors in lymph node metastatic states (105).

## 6 Possible treatment strategies

### 6.1 Exosomes

Investigations into the role of TRIM family proteins in immunotherapy for tumors concentrate on immune checkpoint blockade (ICB), where checkpoint inhibitors, such as anti-programmed cell death-1 (PD-1), demonstrate significant antitumor efficacy (106). TRIM family proteins can also facilitate immune escape through exosomes (107). Exosomes are 30–150 nm-sized extracellular vesicles, originating from primary endosomes through plasma membrane invagination. The plasma membrane contains various bioactive substances and regulates diverse biological processes between cells. Studies confirm that TRIM3 depletion enhances gastric cancer aggressiveness and metastasis, while exosomes harboring TRIM3 suppress TRIM3 expression (27). Detection revealed reduced TRIM3 levels in both gastric cancer tissues and serum-derived exosomes (27).

### 6.2 RNA interference

**Table 3** reveals the critical role of interactions between ncRNAs and TRIM proteins in the oncogenesis, progression, and drug resistance of gastric cancer, providing a clear target and mechanistic basis for the application of RNA interference (RNAi) technology. RNA interference specifically silences target gene expression through molecules such as small interfering RNA (siRNA) and short hairpin RNA (shRNA), enabling precise blockade of oncogenic pathways. For the mRNA sequences of oncogenic TRIM proteins like TRIM37 and TRIM14, complementary siRNAs or shRNAs can be designed to ensure specific silencing of the target gene without affecting other TRIM family members with low homology, thereby reducing off-target effects. For instance, silencing TRIM37 can directly block the NF-κB pathway, inhibiting gastric cancer cell proliferation and cisplatin resistance. For lncRNAs such as ASB16-AS1 and ELFN1-AS1, siRNAs targeting their sequences can be designed to suppress their sponge effect on miRNAs. For example, silencing ELFN1-AS1 can relieve the inhibition of miR-211-3p, thereby downregulating TRIM29 and blocking its oncogenic effects. To prevent the degradation of RNAi molecules and ensure their precise delivery to tumor sites, targeted delivery technologies need to be integrated.

### 6.3 PROTACs

TRIM family proteins can mediate tumor immune escape by regulating tumor cells, the tumor microenvironment, and non-tumor cells. In the body’s anti-tumor response, these proteins exhibit dual functional roles: one group inhibits cancer cell proliferation and invasion while enhancing immune recognition and clearance, whereas the other promotes tumor progression by reinforcing cancer cell immune escape mechanisms. Based on their molecular mechanisms, targeting specific TRIM proteins or intervening in their upstream/downstream signaling pathways has become a key direction for tumor immunotherapy. Specific strategies include: developing gene therapies targeting clinically significant TRIM proteins, combining immune checkpoint inhibitors with radiotherapy and chemotherapy to achieve synergistic anti-tumor effects and improve patient prognosis. Additionally, establishing a tumor-specific database incorporating TRIM family protein expression profiles will facilitate in-depth analysis of patient characteristics, promoting the effective translation of TRIM-related targets from basic research to clinical application.

Proteolysis Targeting Chimeras (PROTACs) represent a novel approach in therapy, utilizing the body’s natural protein degradation processes, specifically the ubiquitin-proteasome system (UPS). This strategy aims to selectively identify and eliminate proteins associated with diseases, facilitating the targeting of “undruggable” proteins that cannot be effectively approached by conventional small-molecule drugs (108). PROTACs can simultaneously bind to the protein of interest (POI) and an E3 ubiquitin ligase, forming a ternary complex (E3 ligase-PROTAC-POI) that induces polyubiquitination of the POI, which is subsequently degraded by the 26S proteasome (109).Currently, a TRIM21-based bioPROTAC (biological PROTAC) has been developed, which can degrade endogenous Human antigen R (HuR) (110). The HuR-regulated proteome may counteract the oncogenic properties of cancer cells *in vivo*, although this requires further investigation in gastric cancer.

Currently, potent and selective ligands targeting the bromodomain of TRIM24 have failed to elicit effective anti-proliferative responses.

A representative compound, Y08624, a TRIM24/BRPF1 bromodomain-targeted inhibitor, demonstrated significant tumor growth inhibition (TGI = 53%) in prostate cancer mouse models (111). Targeted therapy against TRIM protein domains therefore represents a promising future direction for gastric cancer treatment. Recent studies have indicated that TRIM proteins can regulate autophagy and apoptosis in a multi-pronged manner (112). As opposed to HCC, the correlation between TRIM and the autophagy-lysosomal pathway has not been reported in gastric cancer (113) and may provide a method to degrade target genes (114).

PROTACs technology provides a novel approach for targeted therapy against TRIM proteins in gastric cancer, with its advantage lying in the ability to precisely degrade oncogenic proteins rather than merely inhibiting their functions. However, challenges such as insufficient specificity, low delivery efficiency, and limited preclinical data need to be addressed. TRIM family proteins share similar structures (all containing RING domains), which may lead to PROTACs degrading homologous proteins. Additionally, the functions of some TRIM proteins in gastric cancer remain controversial, requiring clarification of their oncogenic or suppressive roles in specific contexts. Moreover, PROTACs have large molecular weights and poor lipophilicity, making it difficult to penetrate the fibrotic microenvironment of gastric cancer solid tumors and cell membranes.

In the future, efforts can be made to optimize the structure of PROTACs through computer-aided design, enhance delivery by combining with nanocarriers, and achieve multi-target synergy by coordinating with ncRNA regulatory axes, thereby promoting their translation into clinical practice.

## 7 Conclusions and prospects

This review systematically summarizes the multiple regulatory mechanisms and clinical translational value of TRIM family proteins in the oncogenesis and progression of gastric cancer. As an important subclass of ubiquitin ligases containing the RING domain, TRIM proteins regulate malignant phenotypes such as tumor cell proliferation, apoptosis, EMT, and resistance to chemotherapy and radiotherapy by mediating ubiquitination of key signaling molecules.

Nevertheless, further research into the specific function of TRIM in gastric cancer imposes challenges. At present, the study of TRIM proteins is limited to the role of homotypic polyubiquitination, but the targets and functions of heterotypic polyubiquitination are not well understood. Although TRIM proteins are closely related to the tumor microenvironment (TME) and help regulate tumor immune escape, few studies have revealed their immunomodulatory role in gastric cancer from a mechanistic and epidemiological perspective. Furthermore, small molecule inhibitors of TRIM proteins have been developed to treat prostate-limited cancer, but have not received attention in gastric cancer. Additionally, the pharmacokinetics and toxicity of TRIM inhibitors and their effects on gastric cancer growth and survival in animal models need to be rigorously evaluated. In the future, if the complete biological system of the TRIM gene can be constructed, it may provide new ideas for the epigenetic mechanism of TRIMs, and contribute to the translational and clinical development of new detection technologies or targeted drugs. For TRIM proteins with clinical significance, pharmacology, pathophysiology, and other disciplines can be applied collectively to translate the important targets and molecular mechanisms of TRIM proteins to clinical practice. From many perspectives, TRIM proteins hold promising significance in gastric cancer.
